# MLN4924 Synergistically Enhances Cisplatin-induced Cytotoxicity via JNK and Bcl-xL Pathways in Human Urothelial Carcinoma

**DOI:** 10.1038/srep16948

**Published:** 2015-11-23

**Authors:** I-Lin Ho, Kuan-Lin Kuo, Shing-Hwa- Liu, Hong-Chiang Chang, Ju-Ton Hsieh, June-Tai Wu, Chih-Kang Chiang, Wei-Chou Lin, Yu-Chieh Tsai, Chien-Tso Chou, Chen-Hsun Hsu, Yeong-Shiau Pu, Chung-Sheng Shi, Kuo-How Huang

**Affiliations:** 1 Department of Urology, College of Medicine, National Taiwan University, and National Taiwan University Hospital, Taipei, Taiwan; 2 Graduate Institute of Toxicology, College of Medicine, National Taiwan University, Taipei, Taiwan; 3 Graduate Institute of Molecular Medicine, College of Medicine, National Taiwan University, Taipei, Taiwan; 4 Department of Pathology, College of Medicine, National Taiwan University, and National Taiwan University Hospital, Taipei, Taiwan; 5 Department of Oncology, National Taiwan University Hospital, Taipei, Taiwan; 6 Graduate Institute of Clinical Medical Sciences, College of Medicine, Chang Gung University, Taoyuan, Taiwan

## Abstract

Cisplatin-based chemotherapy is the primary treatment for metastatic bladder urothelial carcinoma. However, the response rate is only 40–65%. This study investigated the anti-tumor effect and underlying mechanisms of the combination of cisplatin and the NEDD8-activating enzyme inhibitor MLN4924 in human bladder urothelial carcinoma. The combination of cisplatin and MLN4924 exerted synergistic cytotoxicity on two high-grade bladder urothelial carcinoma cell lines, NTUB1 and T24 (combination index <1). MLN4924 also potentiated the cisplatin-induced apoptosis and activation of caspase-3 and -7, phospho-histone H2A.X and PARP. c-Jun N-terminal kinase (JNK) activation and a down-regulation of B-cell lymphoma-extra large (Bcl-xL) were also observed during cisplatin and MLN4924 treatment. Inhibition of JNK activation partially restored cell viability and Bcl-xL expression. Bcl-xL overexpression also rescued cell viability. MLN4924 significantly potentiated cisplatin-induced tumor suppression in urothelial carcinoma xenograft mice. In summary, MLN4924 synergistically enhanced the anti-tumor effect of cisplatin via an increase in DNA damage, JNK activation and down-regulation of Bcl-xL in urothelial carcinoma cells. These findings provide a new therapeutic strategy for the treatment of bladder cancer.

Urinary bladder cancer is estimated to be the sixth most common cancer in the United States, with approximately 74,000 predicted new cases in 2015^1^. Urothelial carcinoma accounts for more than 90% of bladder cancers. The 5-year survival rate for non-invasive and low-grade bladder urothelial carcinoma is approximately 80%, the survival rate is drastically worse for high-grade and invasive urothelial carcinoma^2^. Approximately 50% of cases of high-grade and muscle-invasive bladder urothelial carcinoma will progress to metastatic diseases with a dismal prognosis despite radical cystectomy. The standard therapy for metastatic bladder urothelial carcinoma is cisplatin-based chemotherapy^3^, but the limited response rate because of chemoresistance and chemotherapy-related adverse effects mitigates its clinical efficacy^4^. Therefore, several new regimens and drugs are under investigation to improve the treatment of bladder urothelial carcinoma^5^.

The development of novel therapeutic strategies relies on the discovery of new drugs and new drug combination regimens^6^. Biological systems possess complex signaling networks to maintain homeostasis and normal functions. Cancer cells also possess complex signaling networks to maintain cancer progression. Several newly discovered anti-cancer agents exhibit limited efficacy or encounter resistance because of crosstalk, redundancy, and anti-target activities, which are required for the integrity of signaling networks and reduce the value and direct effect of the agents^6^,^7^. Therefore, drug combinations that simultaneously target the same or different targets are rational approach to improve the efficacy of cancer treatments^8^. To date, several combination therapeutics are standardized and widely used in clinical treatment^9^.

The balance between protein synthesis and turnover affects various cellular functions. The lysosome-mediated degradation pathway and ubiquitin-proteasome system are two major systems that control protein turnover^10^. Ubiquitin is a 76-amino acid protein that is covalently linked to its targets and subjected to the 26 S proteasome for degradation. The ubiquitin-conjugated pathway consists of a three-step mechanism: ubiquitin-activating enzyme (E1) activates ubiquitin, which is transferred to ubiquitin-conjugating enzyme (E2) and finally conjugated to its target proteins by ubiquitin ligase enzyme (E3)^11^. Neural precursor cell expressed, developmentally down-regulated 8 (NEDD8) is a ubiquitin-like molecule that modulates the activity of a subclass of ubiquitin E3 ligases, the cullin-RING ligases^12^. In a manner analogous to ubiquitination, the NEDD8-conjugated pathway is first activated by NEDD8 activating enzyme, and NEDD8 is conjugated to its substrates with the help of E2 and E3 enzymes^12^. The selective NEDD8 activating enzyme inhibitor MLN4924 was identified as a promising anti-cancer drug in 2009, and it was evaluated in several phase I clinical trials^13^. Disruption of neddylation leads to the accumulation of numerous intracellular proteins, which induce DNA damage responses, autophagy, apoptosis and many abnormal cellular responses^14^ that contribute to cytotoxicity in tumor cells. Accumulated substrates associated with cell cycle progression and cell survival regulation also induce apoptosis in tumor cells^15^. Therefore, we hypothesized that a combination of cisplatin and MLN4924 would be a new strategy for the treatment of bladder urothelial carcinoma. Our results demonstrated that the combination of cisplatin and MLN4924 synergistically enhanced the cytotoxicity of cisplatin through increased DNA damage and JNK activation and the down-regulation of the anti-apoptotic protein Bcl-xL *in vitro* and *in vivo*. Our data suggest that the combination of cisplatin and MLN4924 is a high efficacy regimen for the clinical treatment of bladder urothelial carcinoma.

## Results

### Cisplatin and MLN4924 combination synergistically inhibits cell viability in human urothelial carcinoma cells

**Figure 1:**
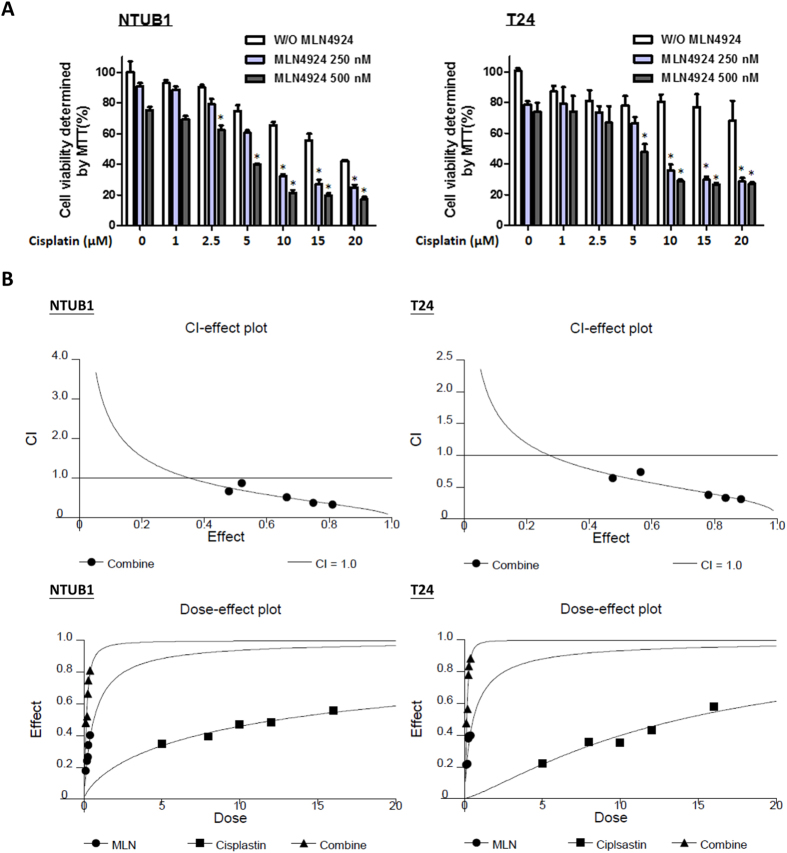
Cisplatin combined with MLN4924 elicits a synergistic effect on the inhibition of cell viability in human urothelial carcinoma cells NTUB1 and T24. (**A**) Human urothelial carcinoma cells NTUB1 and T24 were treated with various concentrations of cisplatin (0 to 20 μM) and MLN4924 (250 nM or 500 nM) alone or in combination for 24 hours. Cell viability was determined by MTT assay. Quantitative analyses of cell viability are presented as the means ± SD of three independent experiments. *p < 0.05 represents a significant difference compared to cisplatin alone. (**B**) Urothelial carcinoma cells were incubated in the presence of cisplatin, MLN4924, or the combination at a concentration ratio of 40:1. Cell viability was measured by MTT assay as described in (**A**). The dose-effect plot reflects the dose-effect relationships of cisplatin, MLN4924 and their combination, and the combination index and fractions affected are plotted in a combination index-effect plot.

We previously reported that the treatment of human bladder urothelial carcinoma cells with MLN4924 significantly induced anti-tumor effects *in vitro* and *in vivo*, and MLN4924 exhibited minimal toxicity on normal urothelial cells^16^. Therefore, we examined the anti-tumor efficacy of the combination of MLN4924 and cisplatin in this study. Two high-grade urothelial carcinoma cell lines, NTUB1 and T24, were treated with MLN4924 (250 and 500 nM) and different concentrations of cisplatin (0 to 20 μM) individually or in combination. Figure 1A shows that cisplatin and MLN4924, alone or in combination, suppressed urothelial carcinoma cell viability, and the inhibitory effect was increased when both agents were used in combination.

The combined drug effects were analyzed using CalcuSyn software to further confirm the synergistic effect of cisplatin combined with MLN4924. The combined effects of cisplatin and MLN4924 at a concentration ratio of 40:1 were subjected to median drug effect and combination index analyses, and the combination index-effect plot and dose-effect plot are shown in Fig. 1B. The combination of cisplatin and MLN4924 exhibited synergistic effects (combination index <1) in NTUB1 and T24 cells, which indicates that the cooperation of both agents increased the inhibitory effect on cell viability.

### Cisplatin and MLN4924 combination induces significant apoptosis and DNA damage in human urothelial carcinoma cells

**Figure 2:**
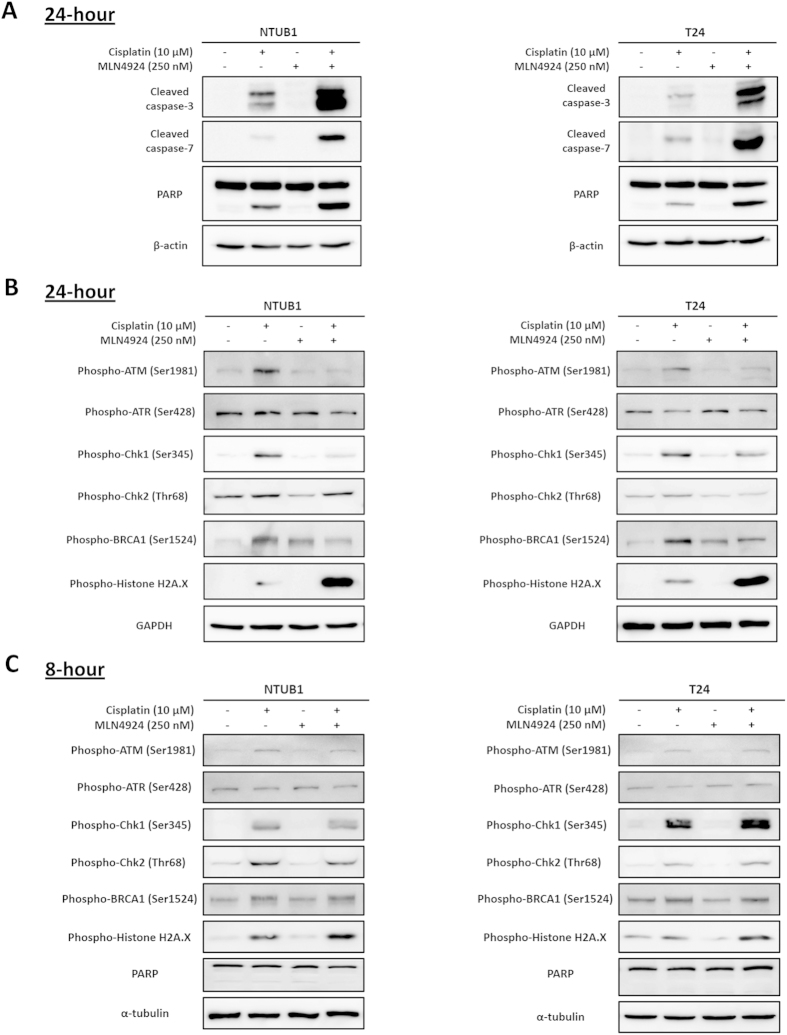
Cisplatin combined with MLN4924 increases apoptosis and DNA damage in human urothelial carcinoma cells NTUB1 and T24. (**A,B**) Human urothelial carcinoma cells NTUB1 and T24 were treated with cisplatin or MLN4924 alone or in combination for 24 hours. Cell lysates were harvested, and the expression level of apoptosis-related molecules (**A**) and DNA damage response regulators (**B**) were assessed using western blotting. (**C**) NTUB1 and T24 cells were treated with cisplatin or MLN4924 alone or in combination for 8 hours. Cell lysates were harvested, and the expression levels of DNA damage response regulators were assessed using western blotting. The results shown are representative of at least three independent experiments. Full-length blots are presented in Supplementary information Fig. S5–S7.

We chose 10 μM cisplatin and 250 nM MLN4924 as our working concentrations for the following investigation. The de-neddylation of cullin-1 in NTUB1 and T24 cells was investigated to ensure that MLN4924-mediated neddylation inhibition was involved in our study (Fig. S1, Supplementary information). A decrease in cell viability may be achieved via apoptosis induction or anti-proliferation alone or in combination. The anti-tumor effect of cisplatin is primarily derived from increased apoptosis^17^, Therefore, we investigated whether the combination of cisplatin and MLN4924 treatment increased apoptosis in NTUB1 and T24 cells. Figure 2A shows increased protein levels of cleaved caspase-3 and -7 and cleaved PARP, which indicates that cisplatin alone and in combination treatment with MLN4924 induced apoptosis in urothelial carcinoma cells. The treatment combination enhanced the apoptotic effects on urothelial carcinoma cells compared to cisplatin alone.

We investigated the underlying mechanisms of these findings. Cisplatin-induced apoptosis occurs primarily through the induction of DNA damage. Several studies examined the combination of MLN4924 with DNA interstrand cross linkage-inducing agents^18^,^19^,^20^. One study reported that the combination of MLN4924 and cisplatin suppressed DNA damage-induced Chk-1 phosphorylation and inhibited DNA repair^21^. We observed that phosphorylated protein levels of ATM, Chk1 and BRCA1, which are involved in the DNA damage response, decreased further after cisplatin and MLN4924 co-treatment. Consistently, the expression of phospho-histone H2A.X, which is a marker of DNA damage, increased with co-treatment compared with cisplatin treatment alone (Fig. 2B). We collected NTUB1 and T24 cell lysates for western blotting 8 hours after cisplatin or MLN4924 alone or in combination before apoptosis occurred to confirm the phosphorylation of histone H2A.X was due to DNA damage and was not induced by apoptotic DNA fragmentation^21^ (Fig. 2C). Phospho-histone H2A.X after cisplatin treatment was observed prior to the onset of apoptosis (i.e., no PARP cleavage). We also found the MLN4924 further increased cisplatin-induced phospho-histone H2A.X before apoptosis occurred, which is consistent with our hypothesis. However, we did not observe a similar trend in phospho-ATM, phospho-Chk1 or phospho-BRCA1 at 8 hours or 24 hours, which indicates that other factors may be responsible for increased DNA damage at early time points. Our data suggest that combination treatment with cisplatin and MLN4924 significantly induced DNA damage and the subsequent apoptosis.

### JNK activation and Bcl-xL down-regulation are implicated in the apoptosis induced by the combination of cisplatin and MLN4924

**Figure 3:**
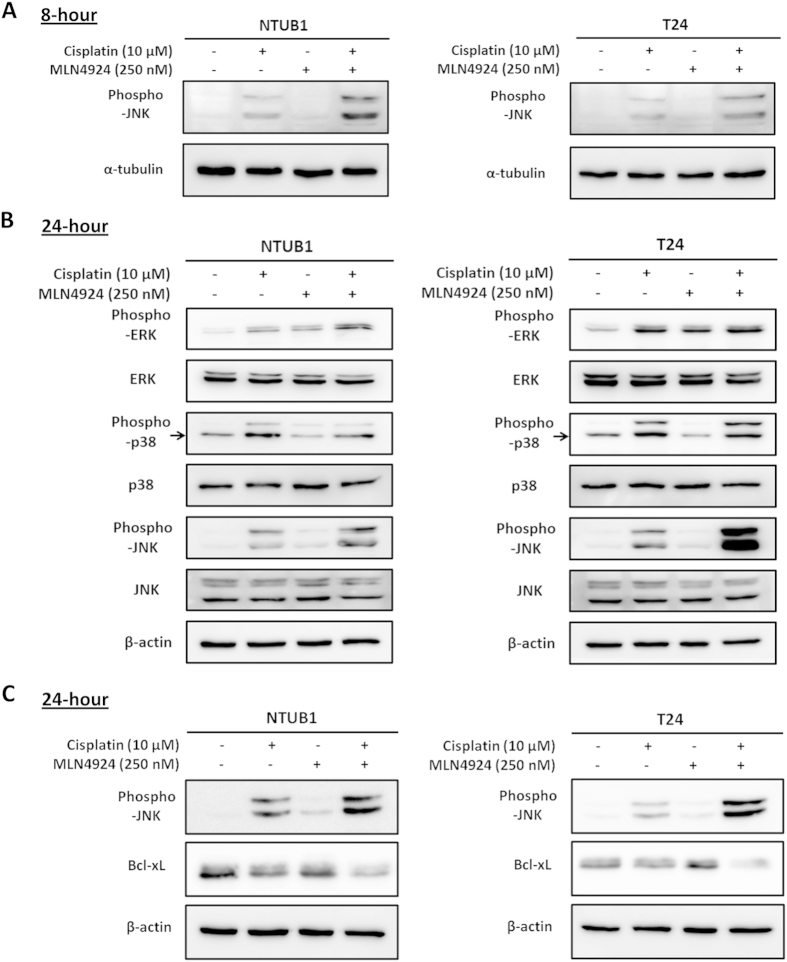
The addition of MLN4924 to cisplatin increases JNK activation simultaneous with Bcl-xL down-regulation. (**A**) Human urothelial carcinoma cells NTUB1 and T24 were treated with cisplatin or MLN4924 alone or in combination for 8 hours. Cell lysates were harvested, and the activation of JNK was assessed using western blotting. (**B,C**) NTUB1 and T24 were treated with cisplatin or MLN4924 alone or in combination for 24 hours. Cell lysates were harvested, and the activation of MAPKs (**B**) and the expression of the anti-apoptotic protein Bcl-xL (**C**) were assessed using western blotting. The results shown are representative of at least three independent experiments. Full-length blots are presented in Supplementary information Fig. S8,S9.

We investigated the precise molecular mechanisms associated with the apoptosis induced by combination treatment with cisplatin and MLN4924 in urothelial carcinoma cells. c-Jun N-terminal kinase (JNK) is linked to a wide variety of DNA damage events^22^,^23^, which prompted us to examine whether JNK activation was involved in our results. Figure 3A shows that JNK was activated after cisplatin treatment alone or in combination with MLN4924 for 8 hours. The level of phospho-JNK was higher in the combined treatment group compared with cisplatin alone. A similar phenomenon was observed after drug treatment for 24 hours (Fig. 3B). These findings suggest that JNK activation participates in apoptosis induced by combined cisplatin and MLN4924 treatment. JNK is in the mitogen-activated protein kinases (MAPK) family. Therefore, we investigated the status of two other MAPK pathway members, extracellular signal-regulated kinases 1/2 (ERK1/2) and p38 kinase^24^. Figure 3B shows that cisplatin and MLN4924 exerted a combined effect only on JNK activation but not ERK1/2 and p38 pathways.

We examined several downstream molecules of the JNK pathway to further elucidate the underlying mechanism of the role of JNK activation in apoptosis^25^,^26^,^27^,^28^. The expression of the anti-apoptotic protein Bcl-xL decreased significantly after cisplatin and MLN4924 co-treatment compared to cisplatin alone (Fig. 3C). Notably, the altered expression level of Bcl-xL was consistent with JNK activation and the occurrence of apoptosis.

### JNK inhibition alleviates apoptosis induced by combined cisplatin and MLN4924 treatment and restores the expression level of Bcl-xL

**Figure 4:**
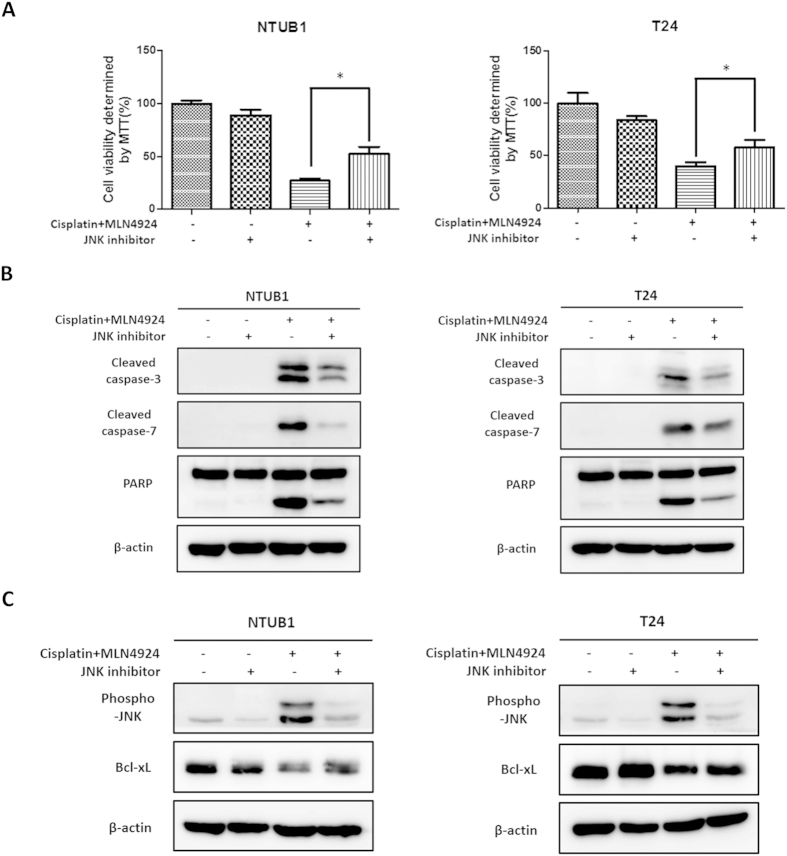
The addition of the JNK inhibitor halts the apoptosis induced by the combination of cisplatin and MLN4924 and reverses the down-regulation of Bcl-xL. (**A**) Human urothelial carcinoma cells NTUB1 and T24 were pretreated with or without a JNK inhibitor (25 μM) for 45 minutes, followed by treatment with the combination of cisplatin (10 μM) and MLN4924 (250 nM) for 24 hours. Cell viability was determined by MTT assay. Quantitative analyses of cell viability are presented as the means ± SD of three independent experiments. *p < 0.05 represents a significant difference compared to the combined groups with or without the JNK inhibitor. (**B,C**) Cell lysates in the same condition as described in (**A**) were collected, and the activation of apoptosis (**B**) and JNK activation and Bcl-xL expression levels (**C**) were investigated using western blotting. The results shown are representative of at least three independent experiments. Full-length blots are presented in Supplementary information Fig. S10.

We inhibited JNK activation to verify our observations. The JNK inhibitor SP600125 competes with ATP and selectively blocks JNK activation^29^. Figure 4A shows that the addition of SP600125 significantly rescued the decreased cell viability caused by the combination of cisplatin and MLN4924 treatment in NTUB1 and T24 cells. This finding suggests that the combinative cytotoxicity of cisplatin and MLN4924 relies on activation of the JNK pathway.

The expression levels of apoptosis-related proteins, including cleaved caspase-3 and -7 and cleaved PARP, were assessed using western blotting. SP600125 treatment significantly decreased apoptosis induced by the combination of cisplatin and MLN4924 (Fig. 4B). We also found that SP600125 partially restored the down-regulation of Bcl-xL in NTUB1 and T24 cells after combined cisplatin and MLN4924 treatment (Fig. 4C).

We knocked down JNK1 using siRNA to examine the existence of off-target effects of SP600125. JNK1 knockdown significantly rescued cell viability, reduced apoptosis and restored Bcl-xL expression levels in NTUB1 and T24 cells (Supplementary information, Fig. S2). These findings confirm our hypothesis that the combination of cisplatin and MLN4924 treatment induces JNK activation and downstream Bcl-xL down-regulation, which leads to apoptosis in urothelial carcinoma cells.

### Bcl-xL overexpression attenuates the apoptosis induced by combination of cisplatin and MLN4924 treatment and exerts no effect on JNK activation

**Figure 5:**
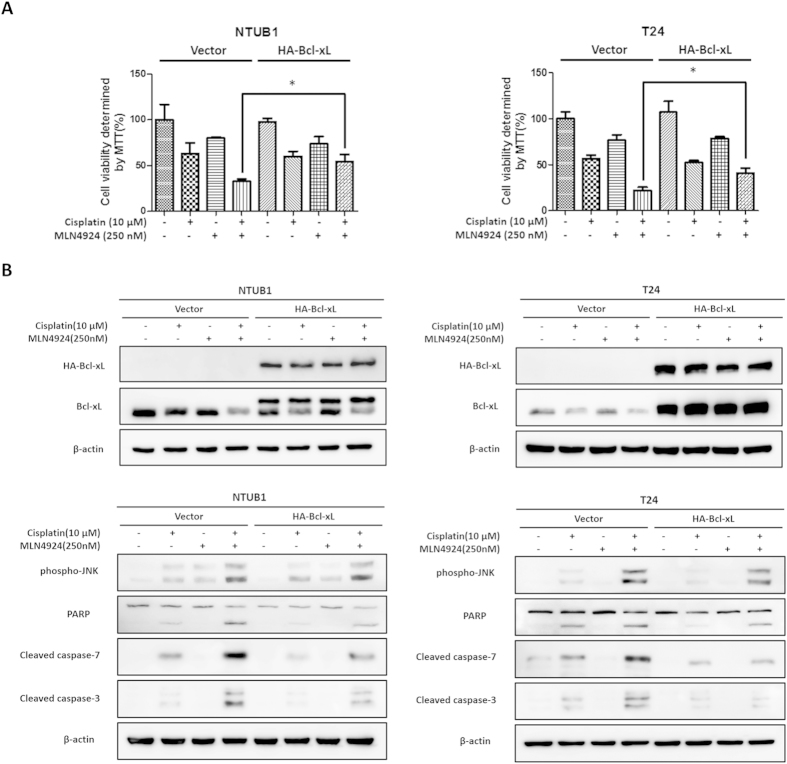
Bcl-xL overexpression attenuates the apoptosis induced by the combination of cisplatin and MLN4924 without influencing JNK activation. (**A**) Human urothelial carcinoma cells NTUB1 and T24 ectopically expressing vector only or HA-Bcl-xL were treated with cisplatin or MLN4924 alone or in combination for 24 hours. Cell viability was determined by MTT assay. Quantitative analyses of cell viability are presented as the means ± SD of three independent experiments. *p < 0.05 represents a significant difference between the indicated groups. (**B**) Cell lysates in the same condition described in (**A**) were collected and analyzed for HA-Bcl-xL expression level, JNK activation and apoptosis activation using western blotting. The results shown are representative of at least three independent experiments. Full-length blots are presented in Supplementary information Fig. S11.

The above findings revealed that the JNK-Bcl-xL axis plays an important role in the apoptosis induced by combination of cisplatin and MLN4924 treatment. We ectopically expressed HA-tagged Bcl-xL in NTUB1 and T24 cells to further confirm our findings. Ectopic expression was achieved using transient transfection of the pcDNA3.0-HA-Bcl-xL plasmid into T24 and NTUB1 cells. Urothelial carcinoma cells with Bcl-xL overexpression exhibited a restored cell viability after combined cisplatin and MLN4924 treatment compared to the control (vector) group (Fig. 5A).

We examined the expression levels of overexpressed Bcl-xL, phospho-JNK and apoptosis-related proteins using western blotting. We measured the protein expression of HA-Bcl-xL to confirm successful overexpression (Fig. 5B) and found that HA-Bcl-xL overexpression suppressed the activation of caspase-3 and -7 and PARP cleavage, but it did not influence JNK activation. Taken together, these findings suggest that the JNK-Bcl-xL axis plays an important role in the apoptosis induced by combined cisplatin and MLN4924 treatment. The modulation of the JNK-Bcl-xL pathway is likely to further enhance the anti-tumor efficacy of cisplatin in urothelial carcinoma cells.

### MLN4924 enhances the cisplatin-induced anti-tumor effect in a xenograft mouse model

**Figure 6:**
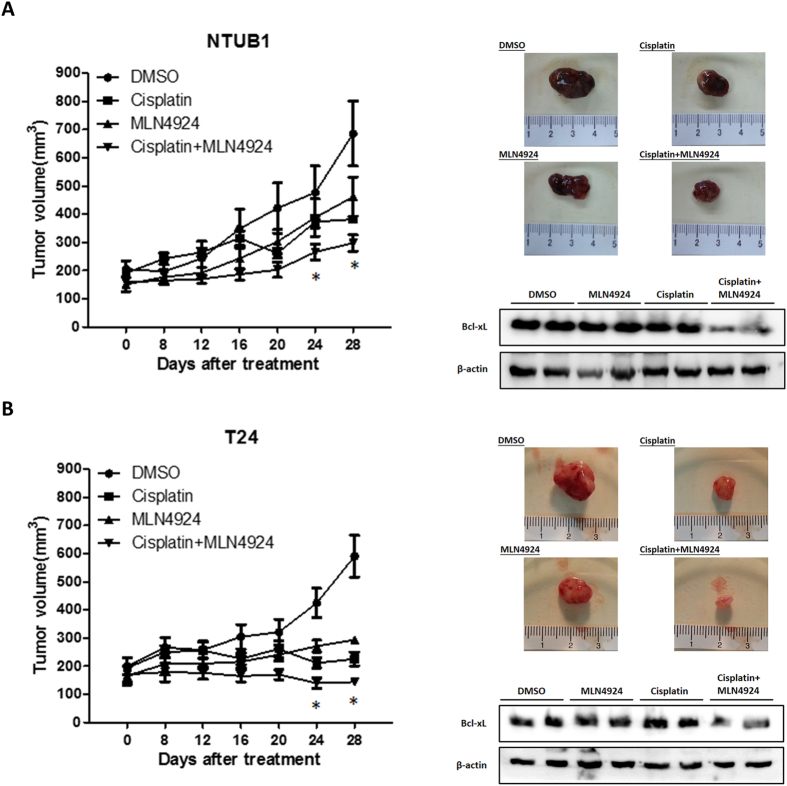
The xenograft model demonstrates the efficacy of the combination of cisplatin and MLN4924 *in vivo*. Nude mice bearing NTUB1 (**A**) or T24 (**B**) xenograft tumors were treated with DMSO (as control), cisplatin, MLN4924 or the cisplatin/MLN4924 combination for 4 weeks. Tumor volumes are presented as the means ± SEM (n = 6 for each group). Tumor images represent excised tumors from each group. The expression level of Bcl-xL in each tumor was assessed using western blotting. *p < 0.05 represents a significant difference between the cisplatin group and the combination group. Full-length blots are presented in Supplementary information Fig. S12.

We evaluated the anti-tumor effects of cisplatin and MLN4924 alone or in combination *in vivo* using a xenograft mouse model. NTUB1 or T24 cells were mixed with Matrigel and injected subcutaneously into the flanks of homozygous null (nu/nu) mice. The mice were divided into four groups (n = 6/group) and received DMSO (non-treated control), cisplatin, MLN4924 or cisplatin/MLN4924 combination intraperitoneally as described in the Methods. The combination of cisplatin and MLN4924 exerted the most significant anti-tumor effect on T24 and NTUB1 xenografts compared to cisplatin or MLN4924 alone (Fig. 6A,B). We examined the expression levels of phospho-JNK and Bcl-xL in xenograft tumor samples from each group to confirm our *in vitro* findings concerning the associations between JNK activation, Bcl-xL down-regulation and apoptosis induced by combination of cisplatin and MLN4924 treatment. Immunohistochemistry was used to quantify JNK activation levels. Figure S3 (Supplementary information) shows that the combination of cisplatin and MLN4924 led to higher JNK activation compared to the single-agent treatment groups. Consistently, western blots revealed decreased Bcl-xL levels after combined cisplatin and MLN4924 treatment in NTUB1 and T24 tumor tissues (Fig. 6A,B). These findings further support the *in vitro* findings that cisplatin and MLN4924 work synergistically to suppress urothelial carcinoma growth via JNK activation and Bcl-xL down-regulation.

## Discussion

Cisplatin was first used for bladder cancer treatment in 1978^30^, and it is still the primary constituent of standard chemotherapeutic regimens. However, its toxicity and the emergence of chemoresistance usually lead to a decline in therapeutic efficacy. Most patients with metastatic urothelial carcinoma rapidly become insensitive to cisplatin treatment, which leads to an ominous prognosis. The precise molecular mechanisms of cisplatin resistance remain unclear, and there is a need to develop novel anti-tumor agents or new drug combinations^6^. Our previous study observed that treatment with MLN4924 elicited significant anticancer effects on bladder urothelial carcinoma^16^. Therefore, we combined MLN4924 with cisplatin for the treatment of urothelial carcinoma in the current study. The effect of drug combinations may be additive, synergistic or antagonistic, which means the effect is equal to, greater than or less than the overall effect of each drug combined^31^. Our results indicated that the combination of cisplatin and MLN4924 was synergistic, which means that the addition of MLN4924 significantly improved the efficacy of cisplatin. We also observed a prominent enhancement of apoptosis in bladder urothelial carcinoma cells under combined cisplatin and MLN4924 treatment, and this effect was reproduced in our *in vivo* model. This *in vivo* result means that the combined treatment improved the therapeutic efficacy compared to cisplatin alone in a clinically relevant manner.

Cisplatin treatment induced the activation of phospho-JNK in the present study. The combination treatment with MLN4924 and cisplatin further increased the activation level of JNK. SAPK/JNK are protein kinases in the MAPK family that are strongly activated in response to stress-related stimuli, such as cytokines, heat shock and oxidative stress^24^. Several previous studies demonstrated that DNA damage induced JNK activation^23^,^32^,^33^. Cisplatin-induced cytotoxicity is associated with its ability to crosslink DNA and induce DNA damage. This DNA damage activates the DNA repair system, and the cell undergoes apoptosis when DNA repair fails^22^. The link between DNA damage and JNK activation led us to investigate the relationship between JNK activation and the combination of cisplatin and MLN4924. The increased expression of the DNA damage marker phospho-histone H2A.X was concurrent with JNK activation during cisplatin treatment alone or in combination with MLN4924, as we anticipated. We suggest that DNA damage plays a role in JNK activation.

A selective NEDD8 activating enzyme inhibitor that disrupts neddylation, MLN4924, is a prominent anti-cancer agent in several cancer types^13^,^14^,^15^. The disruption of neddylation leads to the failure of a major subset of ubiquitin E3 ligase CRLs and the subsequent accumulation of numerous CRL substrates. The combination of MLN4924 and DNA damage agents, such as cisplatin or mitomycin C, enhances DNA damage by interfering with the DNA repair system^18^,^19^. Our study found that cisplatin in combination with MLN4924 suppressed the phosphorylation of ATM, Chk1 and BRCA1 after 24 hours of treatment, and these results were accompanied with a higher expression level of phospho-histone H2A.X. We also found a higher expression level of phospho-histone H2A.X at an early time point (8 hours) of treatment, but a similar trend in phospho-ATM, phospho-Chk1 and phospho-BRCA1 was not observed. The discrepancy between the 8-hour and 24-hour time points may have occurred because MLN4924 manipulated factors that accounted for increased DNA damage at different time points, in other words, MLN4924 may augment DNA damage caused by cisplatin through affecting a set of proteins in early time points, and at later time points, another set of proteins are interfered so that it sustain and increase DNA damage throughout the entire treatment period, which increases JNK activation.

Our results revealed that JNK activation was followed by the down-regulation of Bcl-xL. JNK possesses intrinsic kinase activity and primarily transduces signals via phosphorylation of its downstream substrates. Post-translational modifications, such as phosphorylation, ubiquitination, methylation or SUMOylation, are modifications that lead to conformational changes of the target proteins or provide interaction platforms for the target proteins, which results in the modulation of target protein activity. Previous studies demonstrated that Bcl-2, which is an anti-apoptotic protein similar to Bcl-xL, is phosphorylated on Ser87 and Thr56, and the phosphorylation of Bcl-2 decreases its anti-apoptotic activity, possibly via the inhibition of homo/heterodimer formation, which induces the release of cytochrome c^34^. Similar to Bcl-2, Bcl-xL is phosphorylated on Ser62 by JNK^35^, and other studies reported that the phosphorylation of Ser62 dissociates Bcl-xL from Bax, which allows Bax oligomerization and induces intrinsic apoptotic pathways^36^. We found that JNK activation led to decreased Bcl-xL expression levels. This decrease in Bcl-xL may be the result of a decline in Bcl-xL mRNA or protein levels or both. The 3′UTR of Bcl-xL mRNA binds modulator proteins, such as p38 or nucleolin, to increase mRNA stability^37^,^38^. However, few studies reported the destabilization of Bcl-xL mRNA. Bcl-xL protein turnover occurs via the ubiquitin-proteasome system^39^,^40^. This turnover suggests another possibility that the decreased Bcl-xL protein levels occurred through proteasome-mediated degradation. Ryutaro and colleagues (2014) reported that the Ser62 phosphorylation of Bcl-xL by JNK led to the ubiquitination of Bcl-xL, which was subsequently sent to proteosomes for degradation^28^. We found that Bcl-xL expression level was restored when the proteosome inhibitor MG132 was present during cisplatin and MLN4924 treatment (Supplementary information, Fig. S4). This result demonstrates that the down-regulation of Bcl-xL may occur through proteasome-mediated degradation.

Bcl-xL is a member of the Bcl-2 family that possesses an anti-apoptotic property, and it is critical for cell survival and the progression of malignances^41^,^42^,^43^. There are three subfamilies of the Bcl-2 family that are categorized by the number of BH domains: anti-apoptotic proteins with BH1-4, such as Bcl-2, Bcl-xL, Bcl-w, Mcl-1 and A1/BFL-1; pro-apoptotic proteins with BH1-3, including BAX, BAK, BOK; and the subfamily with only BH3, such as the pro-apoptotic proteins BIK, BID, BIM, BAD, PUMA, NOXA, and HRK^44^. The anti-apoptotic function of Bcl-xL occurs via its ability to inhibit BAX and BAK to halt cytochrome c release. Once death signals, such as stress or growth factor deprivation, activate the BH3-only proteins, BAX and BAK are repressed, which induces intrinsic apoptotic pathways^45^. Our results indicate that the inhibition of JNK activation subsequently rescued the decreased Bcl-xL levels. Bcl-xL overexpression partially reduced the apoptosis caused by the combination of cisplatin and MLN4924. Our *in vivo* model also revealed that the higher JNK activation and significant decline in Bcl-xL protein levels after combined cisplatin and MLN4924 treatment corresponded to significant tumor retardation. Our results demonstrated that Bcl-xL was a prominent target of the combined cisplatin and MLN4924 treatment.

In summary, MLN4924 enhances the cytotoxicity of cisplatin synergistically via increased DNA damage and JNK activation, which represses Bcl-xL expression levels *in vitro* and *in vivo*. These results may provide new strategies in the treatment of bladder cancer.

## Methods

### Cell lines

Two high-grade human bladder urothelial carcinoma cell lines, NTUB1 and T24, were used in this study. The NTUB1 cell line (a gift from Dr. Yeong-Shiau Pu, Department of Urology, National Taiwan University Hospital, Taipei, Taiwan) was derived from a surgical specimen of a 70-year-old female patient with high-grade transitional cell carcinoma, and it was previously demonstrated to be tumorigenic in an animal model^46^,^47^,^48^. The authentication of the NTUB1 cell line has been proven using short tandem-repeat (STR) DNA typing. The T24 cell line was derived from a patient with grade III bladder carcinoma, and it was obtained from the Bioresource Collection and Research Center (BCRC, Hsinchu, Taiwan). The cell lines were cultured in RPMI-1640 medium (NTUB1) and Dulbecco’s Modified Eagle Medium (T24) supplemented with 10% heat-inactivated fetal bovine serum (HyClone, Logan, UT, USA), 1 mM sodium pyruvate (Invitrogen, Carlsbad, CA, USA) and penicillin (100 units/ml)/streptomycin (100 μg/ml) (Invitrogen) at 37 °C with 5% CO_2_. All culture media were purchased from Corning (New York, NY, USA).

### Reagents and antibodies

Cisplatin was obtained from clinical preparations of Abiplatin Solution (Pharmachemie BV, GA Haarlem, The Netherlands). MLN4924 was purchased from Millennium Pharmaceuticals (Cambridge, MA, USA). The c-Jun N-terminal kinase (JNK) inhibitor (SP600125) and MG132 were purchased from Merck Millipore (Billerica, MA, USA). All other chemicals were purchased from Sigma-Aldrich (St. Louis, MO, USA). The following antibodies were used for western blot analysis: B-cell lymphoma-extra large (Bcl-xL), PARP, cleaved caspase-3, cleaved caspase-7, phospho-stress-activated protein kinase (SAPK)/JNK (Thr183/Tyr185), phospho-p44/42 MAPK (Erk1/2) (Thr202/Tyr204), phospho-p38 MAPK (Thr180/Tyr182), SAPK/JNK, p44/42 MAPK (Erk1/2), p38 MAPK, phospho-ATR (Ser428), phospho-BRCA1 (Ser1524), phospho-Chk2 (Thr68), phospho-Chk1 (Ser345), phospho-ATM (Ser1981) and phospho-histone H2A.X (Ser139) were obtained from Cell Signaling Technology (Danvers, MA, USA). Antibodies against CUL-1, α-tubulin, β-actin and GAPDH were purchased from Santa Cruz Biotechnology (Santa Cruz, CA, USA).

### Measurement of cell viability

Cell viability was determined using the MTT assay (Sigma-Aldrich). Briefly, the two cell lines (NTUB1 and T24) were seeded with culture medium in 96-well microplates (5000 cells/well) and incubated at 37 °C for 24  hours before drug treatment. The cells were treated with various treatments for 24 hours and incubated with complete medium containing 0.4 mg/ml MTT at 37 °C for 4 hours. The reduced MTT crystals were dissolved in dimethyl sulfoxide (DMSO, Sigma-Aldrich), and the absorbance was detected at 570 nm using a μQuant ELISA plate reader (Biotek, Winooski, VT, USA).

### Ectopic expression of HA-Bcl-xL in NTUB1 and T24 cells

Human cDNA was obtained via conversion of the total mRNA extracted from the T24 cell line to cDNA. The Bcl-xL cDNA fragment was amplified using the forward primer 5′-ATCGGAATTCATGTCTCAGAGCAACCGGGAG-3′ and the reverse primer 5′-GATCTAGATCATTTCCGACTGAAGAGTGAGC-3′ and subsequently subcloned into the EcoRI and XbaI sites of the pcDNA 3.0-HA expression vector (Invitrogen).

Transfections into NTUB1 and T24 cells were performed using Lipofectamine 2000 (Invitrogen) according to the manufacturer’s instructions. Briefly, cells were plated in 10-cm culture dishes one day before transfection to attain 90% confluency on the day of transfection. Equal quantities of plasmid DNA (10 μg) and 20 μl Lipofectamine 2000 were diluted in two separate tubes of 700 μl OPTI-MEM (Invitrogen) for five minutes at room temperature and mixed by the addition of DNA diluents to Lipofectamine 2000. The DNA/Lipofectamine 2000 mixture was incubated at room temperature for 20 minutes then added drop by drop to the cells and mixed gently with the medium to ensure even distribution. The cells were incubated at 37 °C for 24–36 hours before harvesting for other assays.

### Western blotting

The cells were washed with ice-cold PBS after the various treatments and lysed using cell lysis buffer (Cell Signaling Technology) on ice for 15 minutes followed by centrifugation at 14000 rpm for 15 minutes at 4 °C. The clear supernatants were harvested, and protein concentrations were determined using a BCA protein assay (Thermo Scientific Pierce, Rockford, IL, USA). Equal quantities of each sample were resolved on SDS-PAGE and transferred to a PVDF membrane (GE Healthcare, Piscataway, NJ, USA). Membranes were blocked with 5% skim milk or bovine serum albumin (BSA) in TBST for at least 1 hour, followed by incubation with the respective primary antibodies at 4 °C overnight. The membranes were washed three times with TBST for 10 minutes each and incubated at room temperature for 1 hour with horseradish peroxidase-conjugated secondary antibodies (Genetex, Irvine, CA, USA). Antibody-bound membranes were washed twice with TBST and treated with enhanced chemiluminescent western blotting detection reagents (Millipore, Billerica, MA, USA). Protein bands were visualized using an ImageQuant LAS 4000 system (GE Healthcare).

### *In vivo* xenograft experiments

All animal care and experimental procedures were performed in accordance with protocols approved by the National Taiwan University College of Medicine and College of Public Health Institutional Animal Care and Use Committee (IACUC). A total of 48 animals were used in the present study.

A total of 5 × 10^5^ NTUB1 or T24 cells were suspended in 200 μl of serum-free media and mixed with an equal volume of Matrigel (BD Biosciences, Bedford, MA, USA). The mixtures were injected subcutaneously into the dorsal flanks of 8-week-old Nu/Nu mice, which were obtained from BioLASCO Experimental Animal Center (Taipei, Taiwan). After the tumors had grown to approximately 150 mm^3^, the mice were treated with cisplatin, MLN4924, cisplatin combined with MLN4924, or the control group (n = 6 for each group). The cisplatin- and MLN4924-treated groups received intraperitoneal injections of 3 mg/kg cisplatin and 25 mg/kg MLN4924 in normal saline, respectively, three times weekly for four weeks. The combined group received the same doses of both agents at the same frequency and duration. The non-treated control group received a mixture of DMSO and normal saline. Tumor volume was measured every four days using calipers and calculated as follows: volume = longest tumor diameter × (shortest tumor diameter)^2^/2. Tumors were excised after four weeks of treatment and photographed; for each tumor, about half of tumor was frozen in liquid nitrogen and stored at −80 °C, and the rest part was fixed with 10% formalin and embedded in paraffin. Tissue proteins were extracted using the T-PER tissue protein extraction reagent (Thermo Scientific Pierce, Rockford, IL, USA), and protein expression was analyzed using western blotting. The paraffin-embedded tissues were used for immunohistochemical analysis.

### Combined drug effects

The combined effects of cisplatin and MLN4924 were determined using CalcuSyn software (version 1.1.1, 1996, Biosoft, Cambridge, UK). The combined effects at a combination ratio (1:40) were subjected to median-effect analysis as previously described^49^,^50^. We determined the effects of cisplatin and MLN4924 alone, then we measured the effect of drug combination. Numerous combined effects of growth inhibition were obtained by combining the two agents at graded concentrations, and the results were analyzed using CalcuSyn software. A combination index was generated for each combined dose effect (or fraction affected). The effects of the combinations were transformed into fraction-affected combination index plots. Combination index values less than 1, equal to 1, and greater than 1 were defined as synergism, additive, and antagonism, respectively.

### Statistical analysis

GraphPad Prism® 5 software (La Jolla, CA, USA) was used for all data analyses. All data are reported as the means ± SD, except where indicated otherwise. Data of two groups were analyzed using a two-tailed Student’s t-test, and data of multiple groups were analyzed using a one-way ANOVA followed by the Bonferroni post-hoc test. A p value less than 0.05 was considered statistically significant.

## Additional Information

**How to cite this article**: Ho, I.-L. *et al.* MLN4924 Synergistically Enhances Cisplatin-induced Cytotoxicity via JNK and Bcl-xL Pathways in Human Urothelial Carcinoma. *Sci. Rep.* **5**, 16948; doi: 10.1038/srep16948 (2015).

## Supplementary Material

Supplementary Information
