# Supplementary material for: MLN4924 Synergistically Enhances Cisplatin-induced Cytotoxicity via JNK and Bcl-xL Pathways in Human Urothelial Carcinoma
Source: Sci Rep. 2015 Nov 23;5:16948. doi: 10.1038/srep16948 (PMC4655337; doi:10.1038/srep16948)
Supplement: Supplementary Information [file srep16948-s1.pdf]

Supplementary Information

MLN4924 Synergistically Enhances Cisplatin-induced Cytotoxicity via JNK and Bcl-xL Pathways in Human Urothelial Carcinoma

I-Lin Ho[1*], Kuan-Lin Kuo[1,2*], Shing-Hwa Liu[2], Hong-Chiang Chang[1], Ju-Ton Hsieh[1], June-Tai Wu[3], Chih-Kang Chiang[2], Wei-Chou Lin[4], Yu-Chieh Tsai[5], Chien-Tso Chou[1], Chen-Hsun Hsu[1], Yeong-Shiau Pu[1], Chung-Sheng Shi[6‡], Kuo-How Huang[1‡]

Supplementary Figure S1

Supplementary Figure S2

Supplementary Figure S3

Supplementary Figure S4

Supplementary Figure S5

Supplementary Figure S6

Supplementary Figure S7

Supplementary Figure S8

Supplementary Figure S9

Supplementary Figure S10

Supplementary Figure S11

Supplementary Figure S12

Figure S1

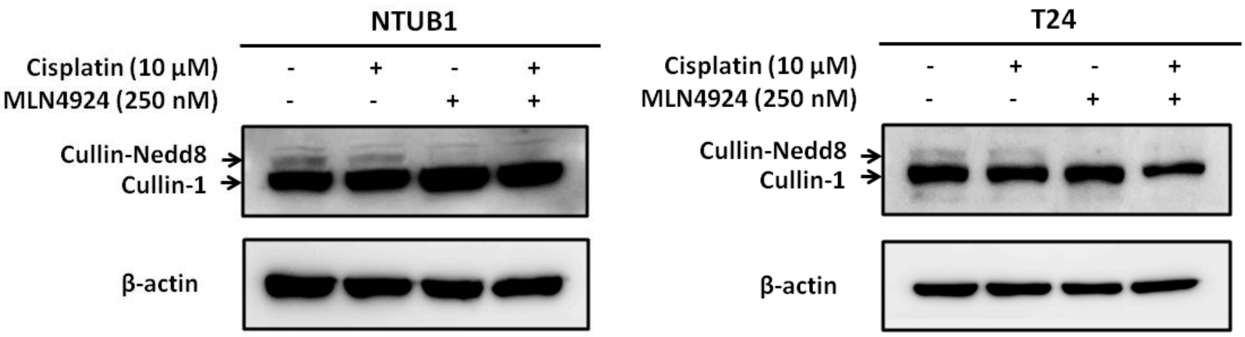

Fig S1. MLN4924 induces de-neddylation in NTUB1 and T24 cells. NTUB1 and T24 cells were treated with cisplatin and MLN4924 alone or in combination for 24 hours. Cell lysates were harvested and Cullin-1 was chosen as the target to demonstrate the neddylation inhibition acticity of MLN4924 via western blotting. The results shown are representative of at least three independent experiments.

Figure S2.

(A)

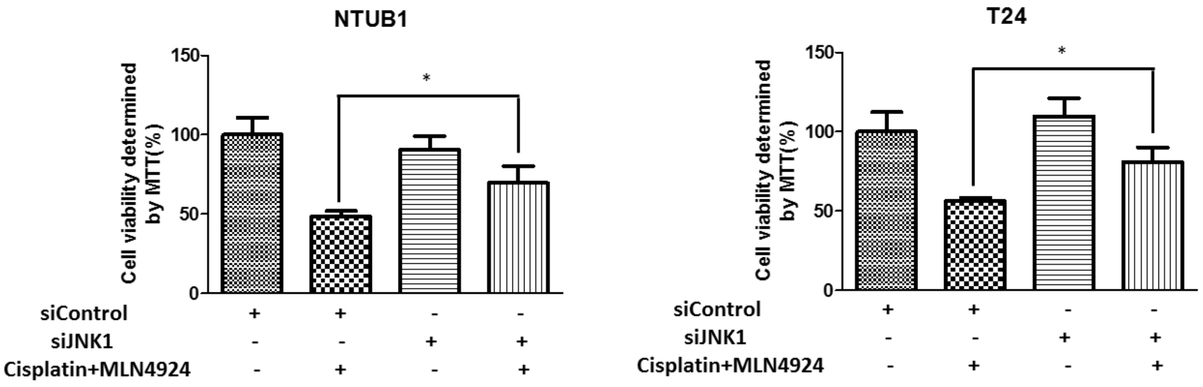

(B)

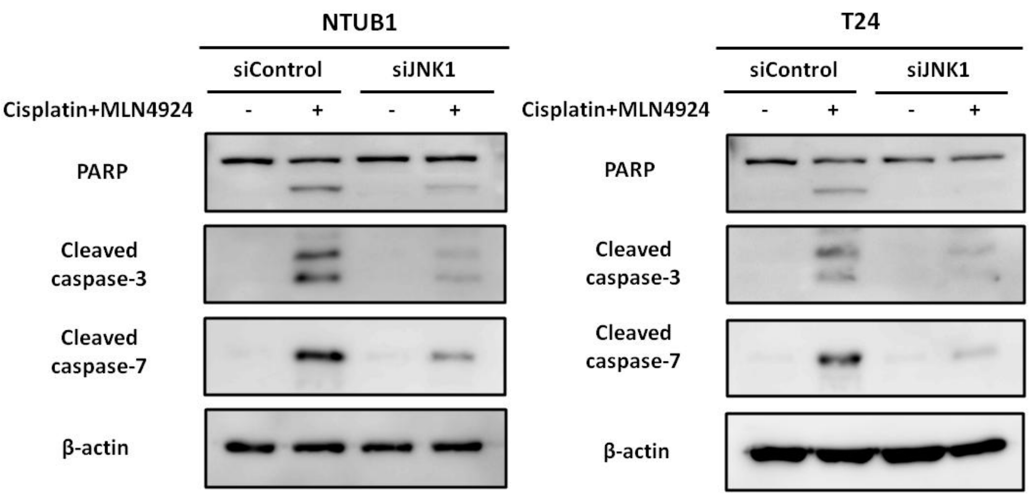

(C)

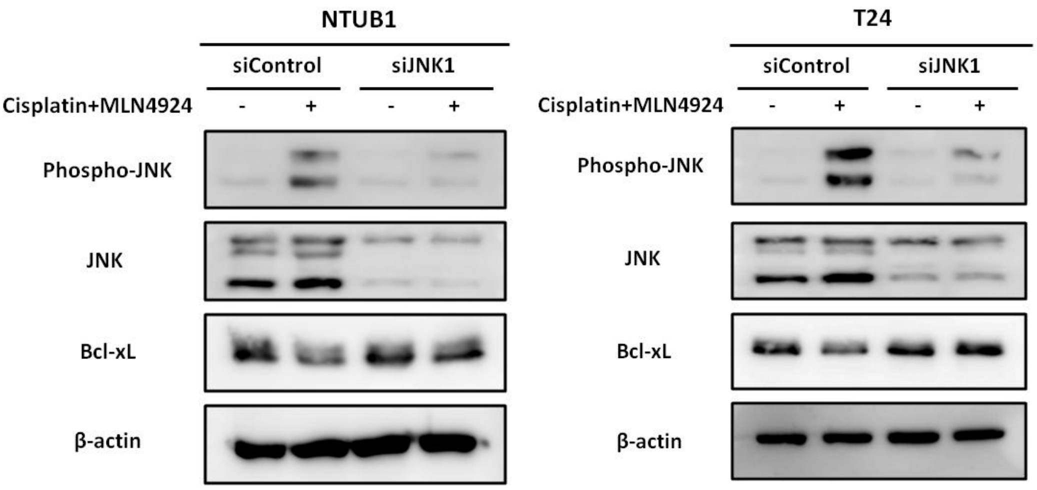

Fig S2. JNK1 knockdown attenuates the apoptosis induced by the combination of cisplatin and MLN4924 and reverses the down-regulation of Bcl-xL. NTUB1 and T24 cells were transfected with 25 nM siRNA mixed with DharmaFECT1 transfection reagent. After incubation for 48 hours, cells were treated with cisplatin (10 μM) and MLN4924 (250 nM) for 24 hours. (A) Cell viability was determined by MTT assay. Quantitative analyses of cell viability are presented as the means ± SD of three independent experiments. *$p<0.05$ represents a significant difference between the indicated groups. (B) and (C) Cell lysates were collected, and the activation of apoptosis (B) and JNK and Bcl-xL expression levels (C) were investigated using western blotting. The results shown are representative of at least three independent experiments.

Fig S3.

(A)

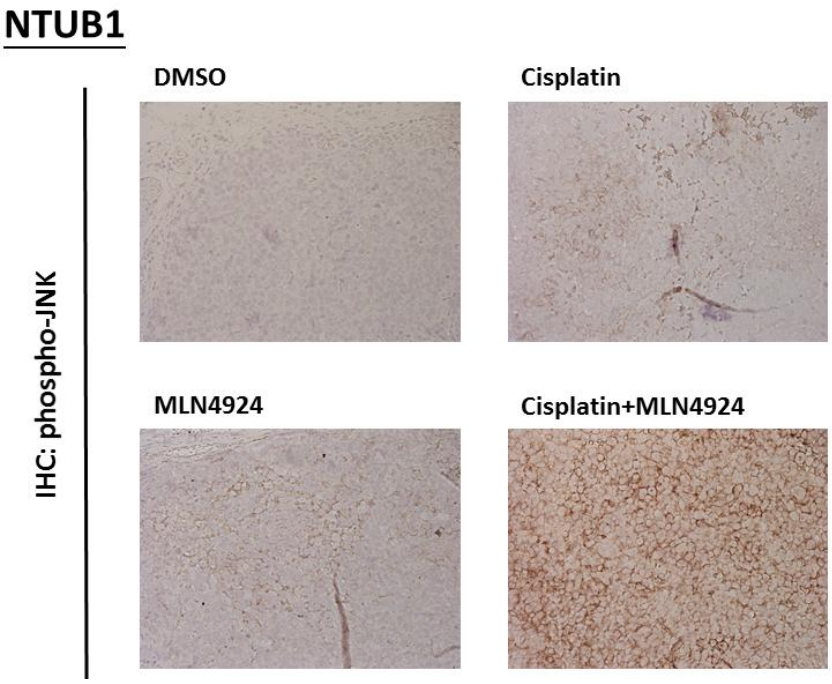
NTUB1
DMSO
Cisplatin
MLN4924
Cisplatin+MLN4924
IHC: phospho-JNK

(B)

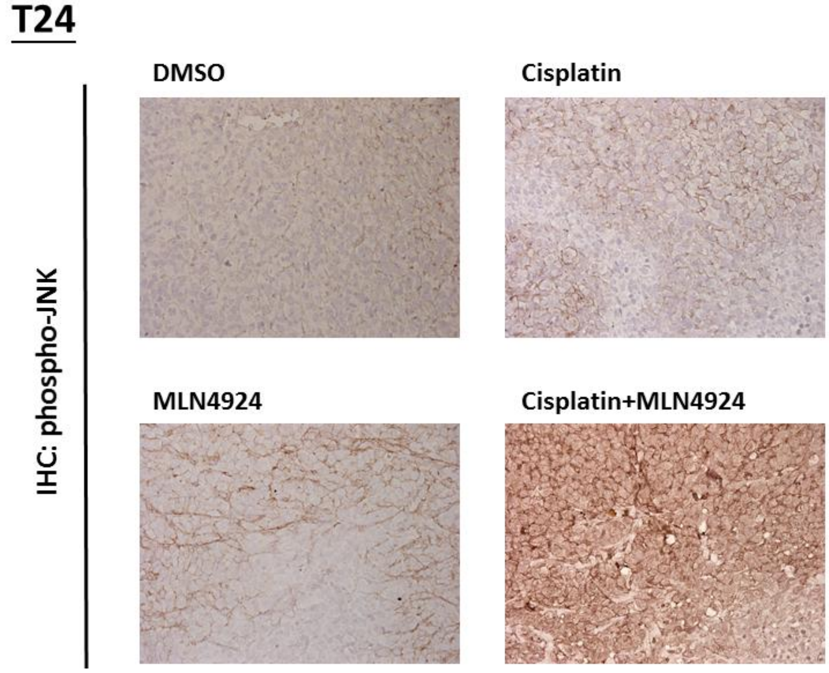
T24
DMSO
Cisplatin
MLN4924
Cisplatin+MLN4924
IHC: phospho-JNK

Fig S3. The combination of cisplatin and MLN4924 treatment induces higher JNK activation in NTUB1 and T24 xnograft tumor tissues.

(A) and (B) Paraffin-embedded tissue sections were deparaffinized in xylene and rehydrated through descending grades of ethanol. After heat-induced antigen retrieval, the endogenous peroxidase activity was quenched by treating the tissues with 3% H2O2 for 10 minutes and rinsed in PBS for 5 minutes. The nonspecific binding was blocked with PBS containing 10% FBS and 3% BSA and incubated at room temperature for 90 minutes. After washing with PBS, slides were incubated with 1:50 dilution of anti- phospho-stress activated protein kinase (SAPK)/JNK (Thr183/Tyr185) primary antibodies (Cell Signaling Technology) at 4 ℃ overnight. Next day, slides were washed twice with PBS and incubated at RT with secondary antibodies (Dako REAL™ EnVision™ Detection System) for 30 minutes. After washing with PBS, the sections were incubated with 3´,3´-diaminobenzidine (DAB) (Dako REAL™ EnVision™ Detection System) for 10 minutes. The tissue sections were then briefly rinsed in PBS, counterstained with hematoxylin (Sigma-Aldrich, St. Louis, MO, USA) and then mounted for microscope visualization (200X).

Figure S4.

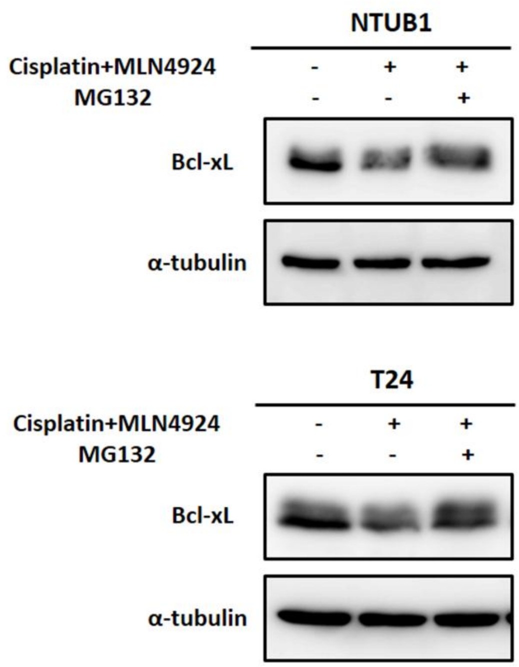

Fig S4. The expression level of Bcl-xL is restored in the presence of MG132 when NTUB1 and T24 cells are treated with cisplatin and MLN4924 combination.

NTUB1 and T24 cells were treated with the combination of cisplatin (10 μM) and MLN4924 (250 nM) with or without the presence of MG132 (10 μM), a proteasome inhibitor for 16 hours. Cell lysates were harvested and the expression level of Bcl-xL was assessed via western blotting. The results shown are representative of at least three independent experiments.

Figure S5.

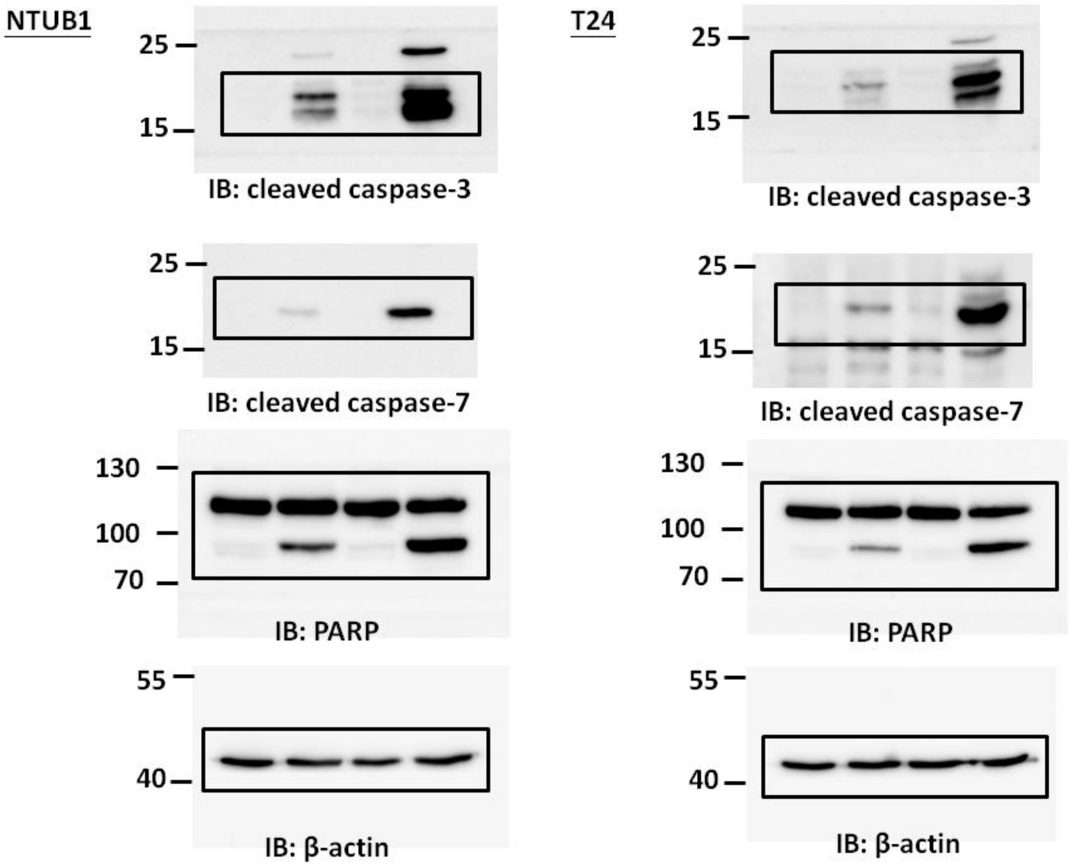

Figure S5. **Full-length gels and blots for main figures corresponded to Fig. 2A.** Molecular weight is shown at the left panel (kDa).

Figure S6.

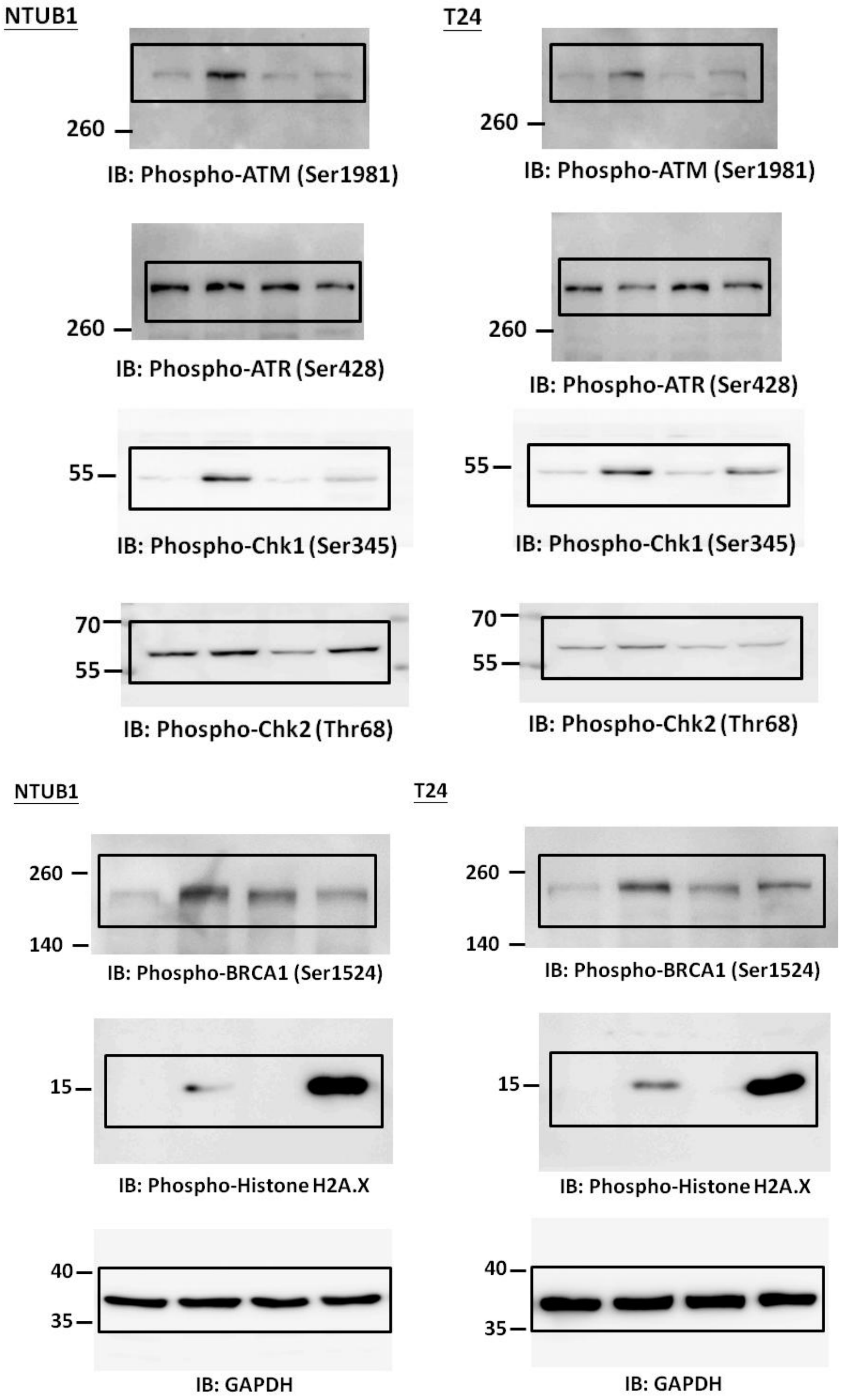

Figure S6. **Full-length gels and blots for main figures corresponded to Fig. 2B.** Molecular weight is shown at the left panel (kDa).

Figure S7.

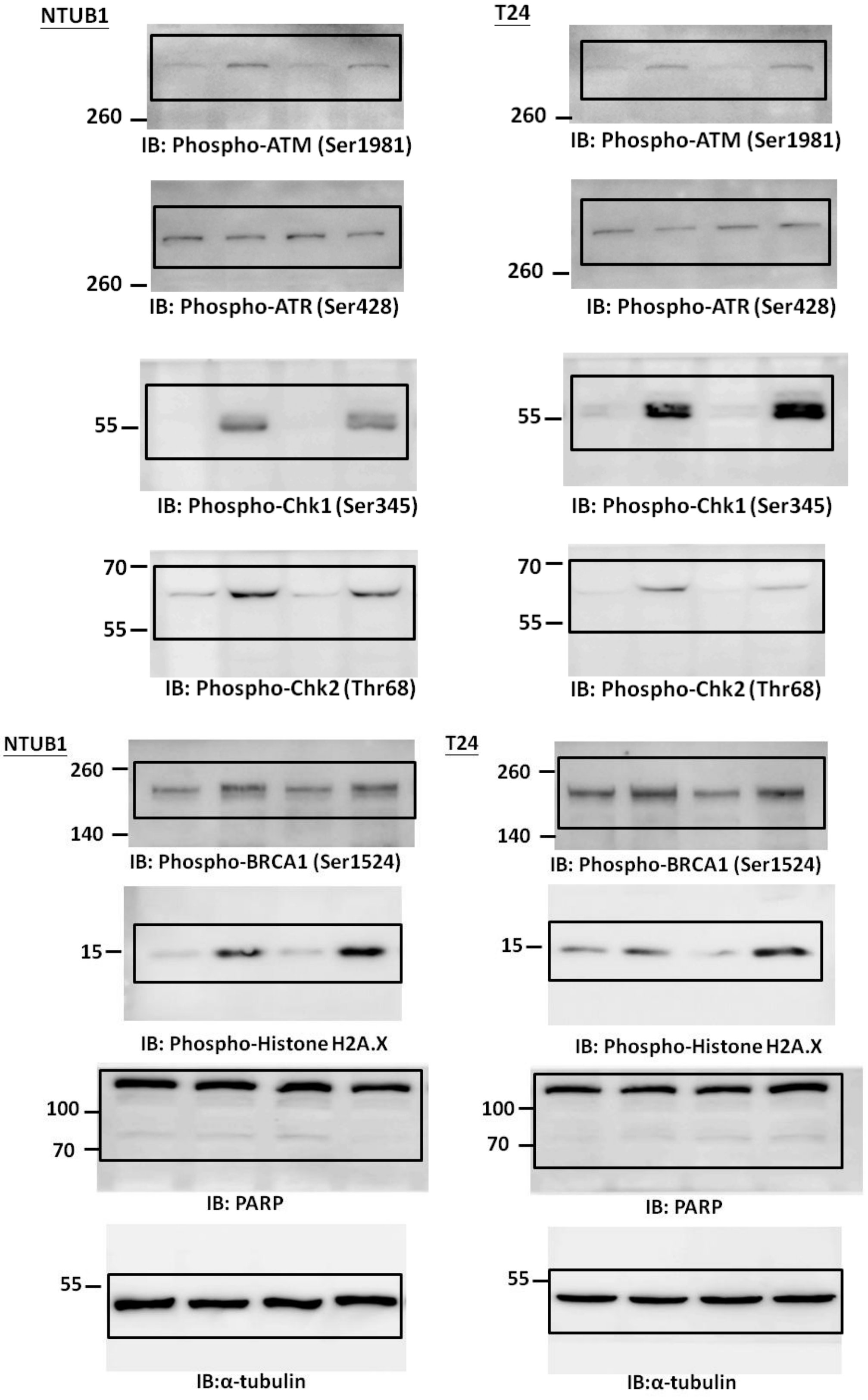

Figure S7. **Full-length gels and blots for main figures corresponded to Fig. 2C.** Molecular weight is shown at the left panel (kDa).

Figure S8.

A

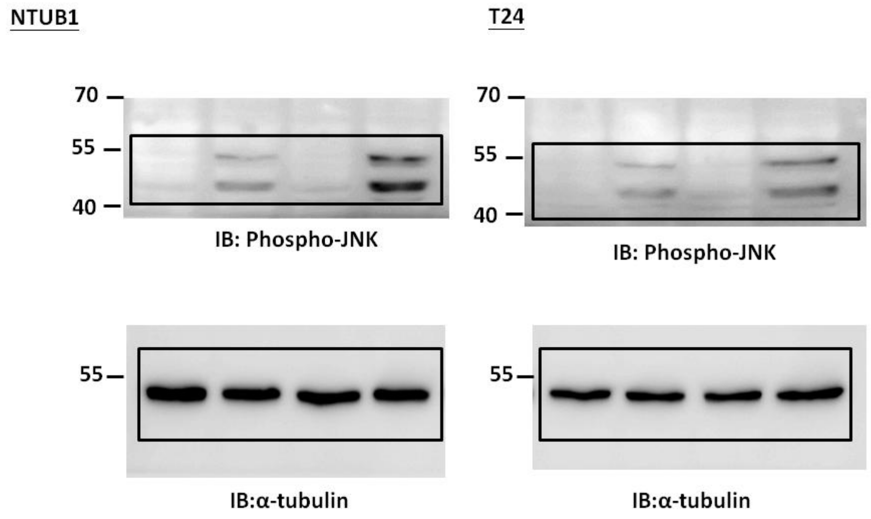

B

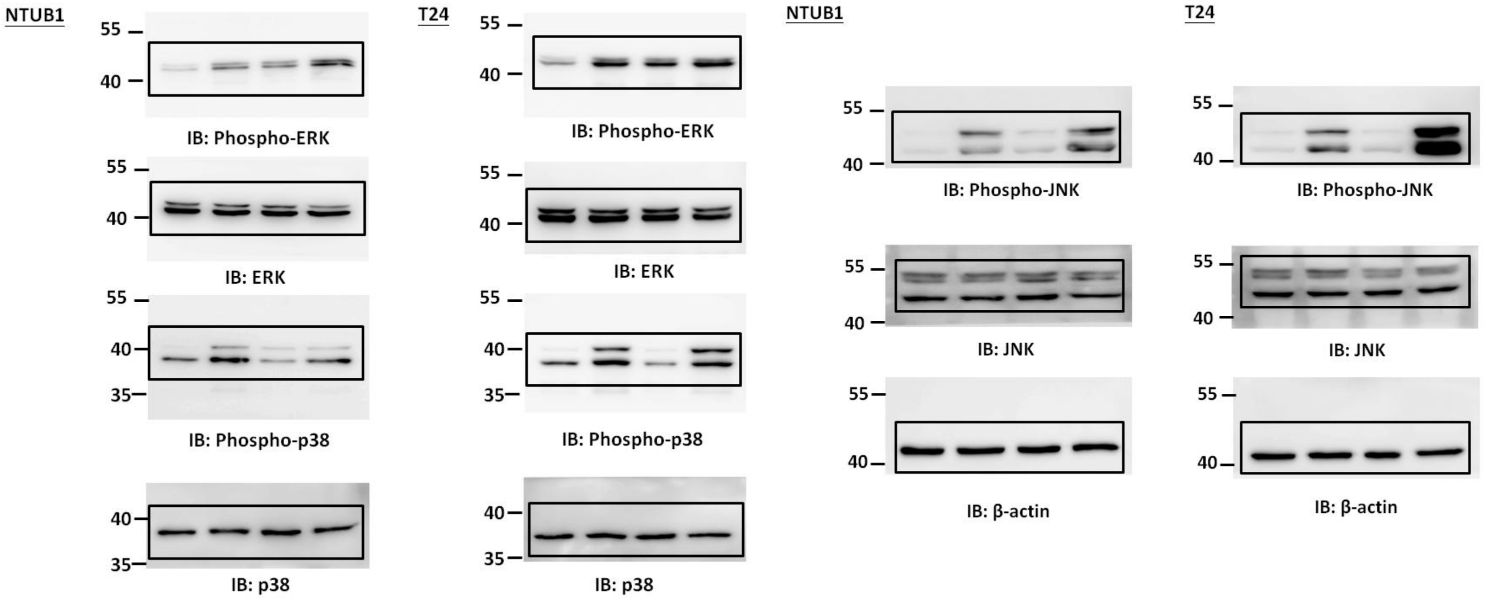

Figure S8. **Full-length gels and blots for main figures corresponded to Fig. 3A and 3B.** Molecular weight is shown at the left panel (kDa).

Figure S9.

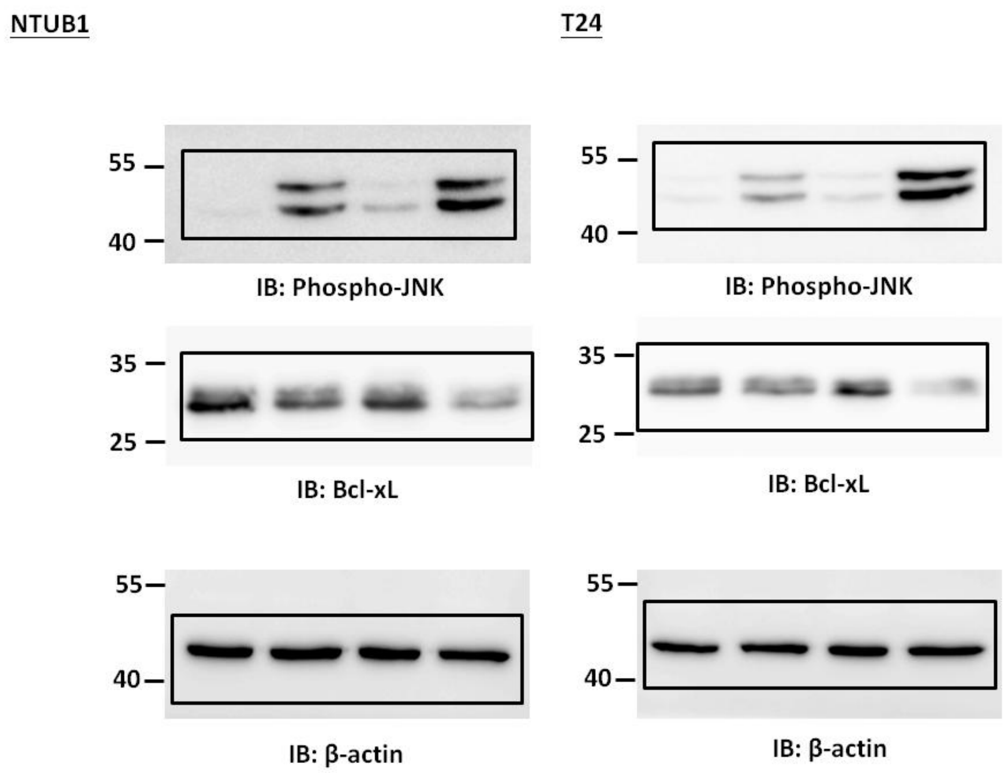

Figure S9. **Full-length gels and blots for main figures corresponded to Fig. 3C.** Molecular weight is shown at the left panel (kDa).

Figure S10.

B

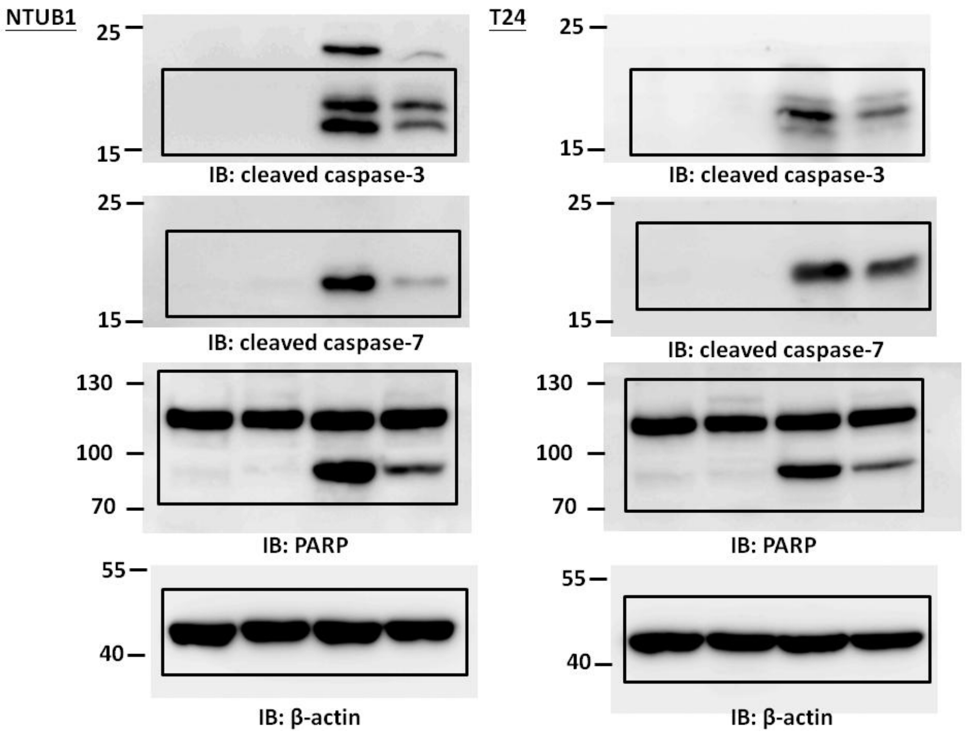

C

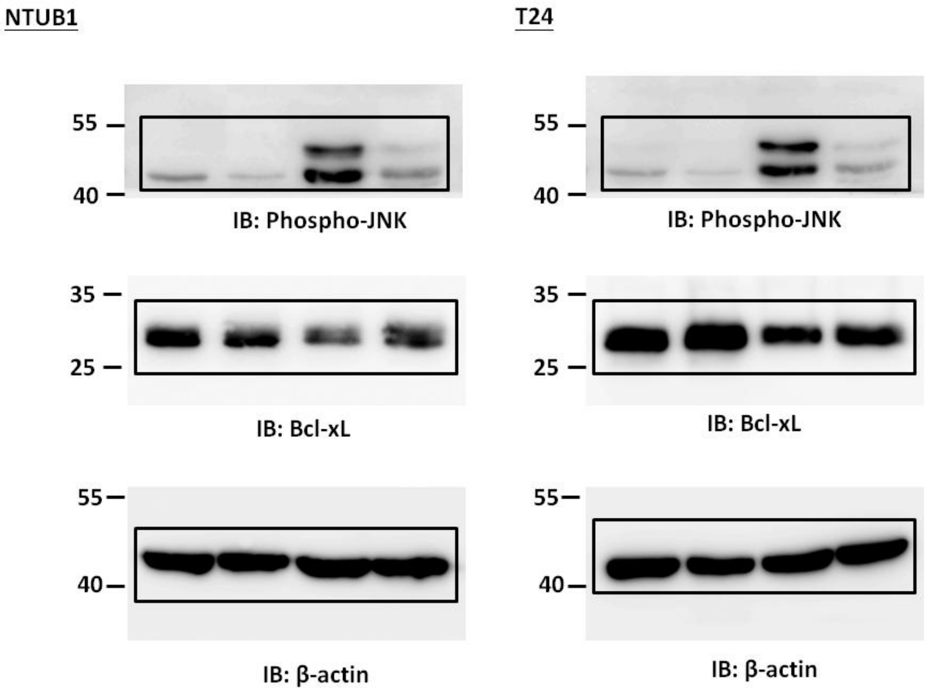

Figure S10. **Full-length gels and blots for main figures corresponded to Fig. 4B and 4C.** Molecular weight is shown at the left panel (kDa).

Figure S11.

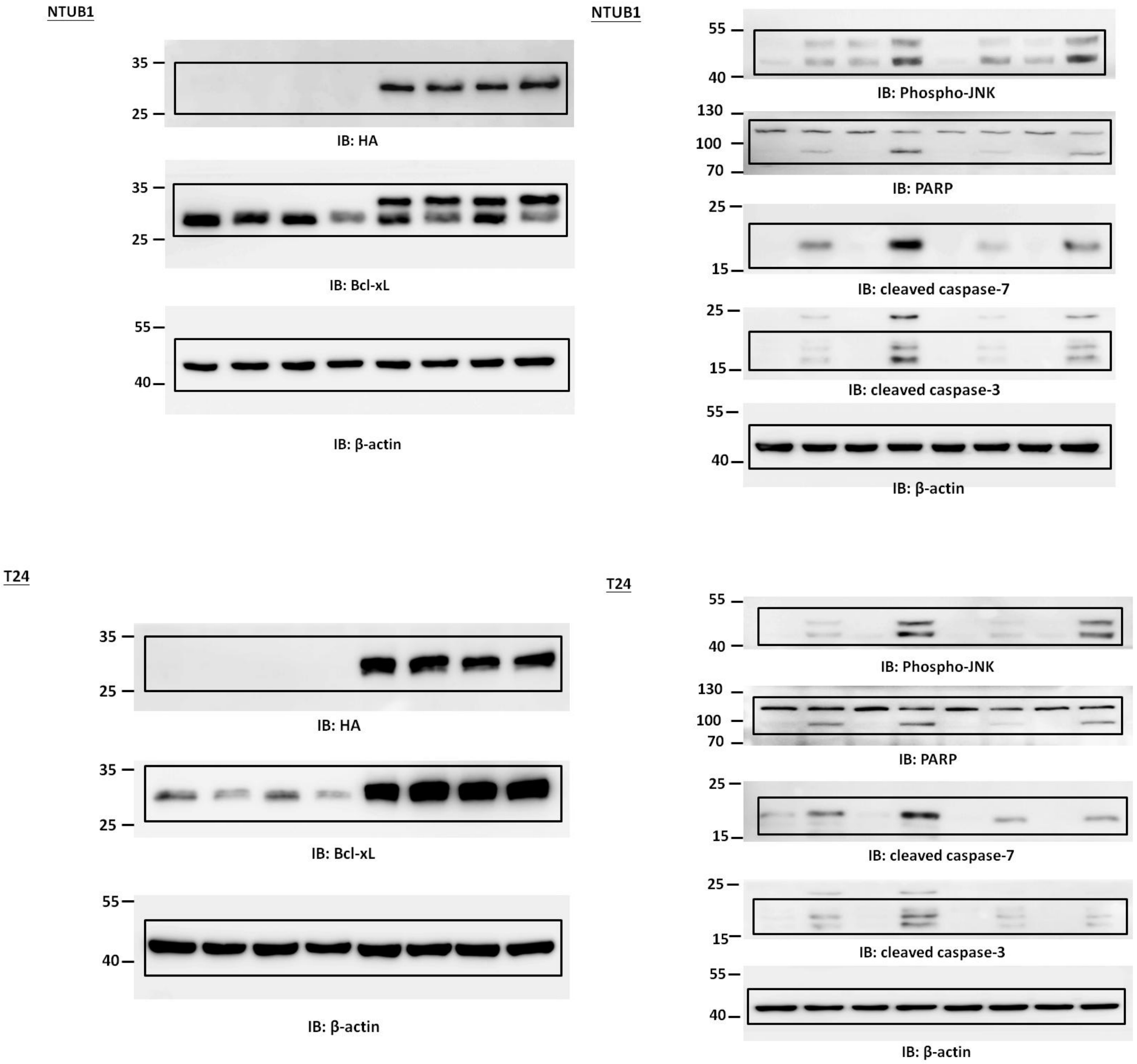

Figure S11. **Full-length gels and blots for main figures corresponded to Fig. 5B.** Molecular weight is shown at the left panel (kDa).

Figure S12.

A

NTUB1

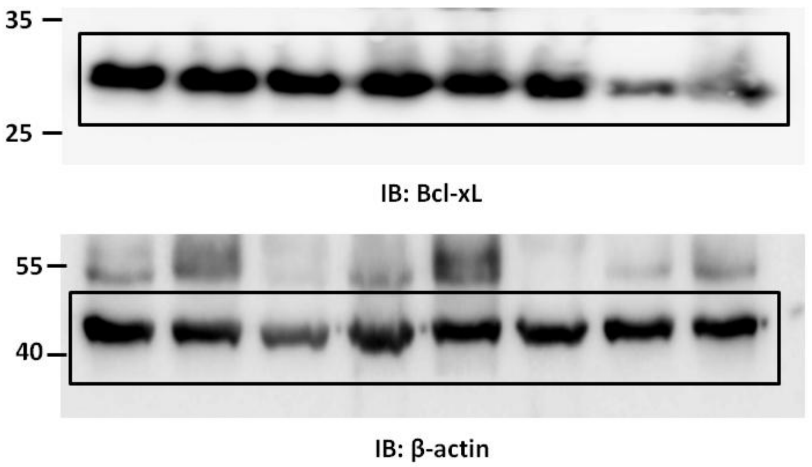

B

T24

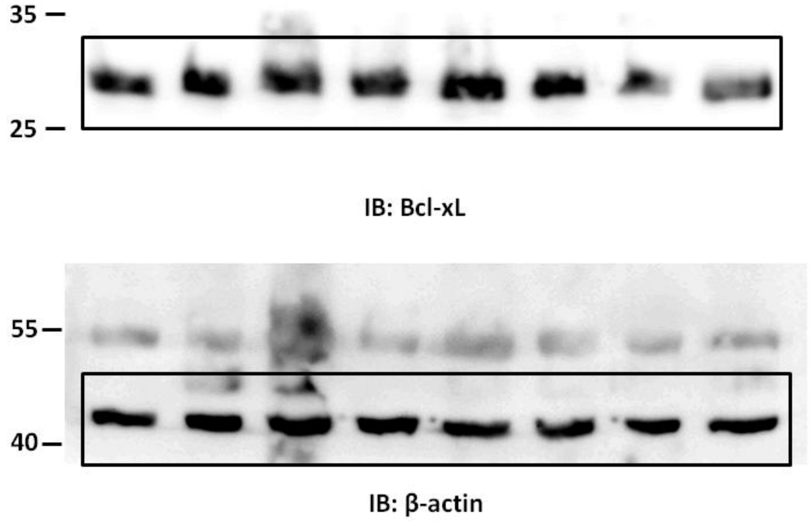

Figure S12. **Full-length gels and blots for main figures corresponded to Fig. 6A and 6B.** Molecular weight is shown at the left panel (kDa).
